# The Nature and Perception of Fluctuations in Human Musical Rhythms

**DOI:** 10.1371/journal.pone.0026457

**Published:** 2011-10-26

**Authors:** Holger Hennig, Ragnar Fleischmann, Anneke Fredebohm, York Hagmayer, Jan Nagler, Annette Witt, Fabian J. Theis, Theo Geisel

**Affiliations:** 1 Max Planck Institute for Dynamics and Self-Organization, Göttingen, Germany; 2 Institute for Nonlinear Dynamics, University of Göttingen, Göttingen, Germany; 3 Department of Physics, Harvard University, Cambridge, Massachusetts, United States of America; 4 Institute of Psychology, University of Göttingen, Göttingen, Germany; 5 Department of Primary Care and Public Health Sciences, King's College, London, United Kingdom; 6 Bernstein Center for Computational Neuroscience (BCCN), Göttingen, Germany; 7 Institute for Bioinformatics and Systems Biology, Helmholtz Zentrum München, Neuherberg, Germany; Tel Aviv University, Israel

## Abstract

Although human musical performances represent one of the most valuable achievements of mankind, the best musicians perform imperfectly. Musical rhythms are not entirely accurate and thus inevitably deviate from the ideal beat pattern. Nevertheless, computer generated perfect beat patterns are frequently devalued by listeners due to a perceived lack of human touch. Professional audio editing software therefore offers a humanizing feature which artificially generates rhythmic fluctuations. However, the built-in humanizing units are essentially random number generators producing only simple uncorrelated fluctuations. Here, for the first time, we establish long-range fluctuations as an inevitable natural companion of both simple and complex human rhythmic performances. Moreover, we demonstrate that listeners strongly prefer long-range correlated fluctuations in musical rhythms. Thus, the favorable fluctuation type for humanizing interbeat intervals coincides with the one generically inherent in human musical performances.

## Introduction

The preference for a composition is expected to be influenced by many aspects such as cultural background and taste. Nevertheless, universal statistical properties of music have been unveiled. On *very long* time scales, comparable to the length of compositions, early numerical studies indicated “flicker noise” in musical pitch and loudness fluctuations [1], [2] characterized by a power spectral density of 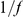 type, 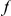 denoting the frequency. In reverse, these findings lead physicists to create so-called *stochastic* musical compositions. Most listeners judged these compositions to be more pleasing than those obtained using uncorrelated noise or short-term correlated noise [1].

Rhythms play a major role in many physiological systems. Prominent examples include wrist motion [3] and coordination in physiological systems [4]–[8]. An enormous number of examples for 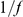-noise in many scientific disciplines, such as condensed matter [9], [10], econophysics [11], and neurophysics [12]–[14] made general concepts explaining the omnipresence of 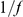-noise conceivable [15]–[19]. However, to mention only a few, studies of heartbeat intervals [20]–[23], gait intervals [7], [24], and human sensorimotor coordination [4]–[6], [8], [25] revealed long-range fluctuations significantly deviating from 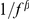 noise with 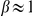. Negative deviations from 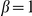 involve fluctuations with weaker persistence than flicker noise which is *superpersistent*. Thus the scaling exponent 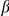 is important for characterizing the universality class of a long-range correlated (physiological) system.

An ancient and yet evolving example of coordination in physiological systems are human musical performances. The neuronal mechanisms of timing in the millisecond range are still largely unknown and subject of scientific research [18], [26]–[31]. However, the nature of temporal fluctuations in complex human musical rhythms has never been scrutinized as yet. In this article, we study the correlation properties of temporal fluctuations in music on the timescale of rhythms and their influence on the perception of musical performances. We found long range correlations for both simple and complex rhythmic tasks and for both laypersons and professional musicians well outside the 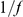 regime. On the other hand, our study unveils a significant preference of listeners for long-range correlated fluctuations in music.

**Figure 1:**
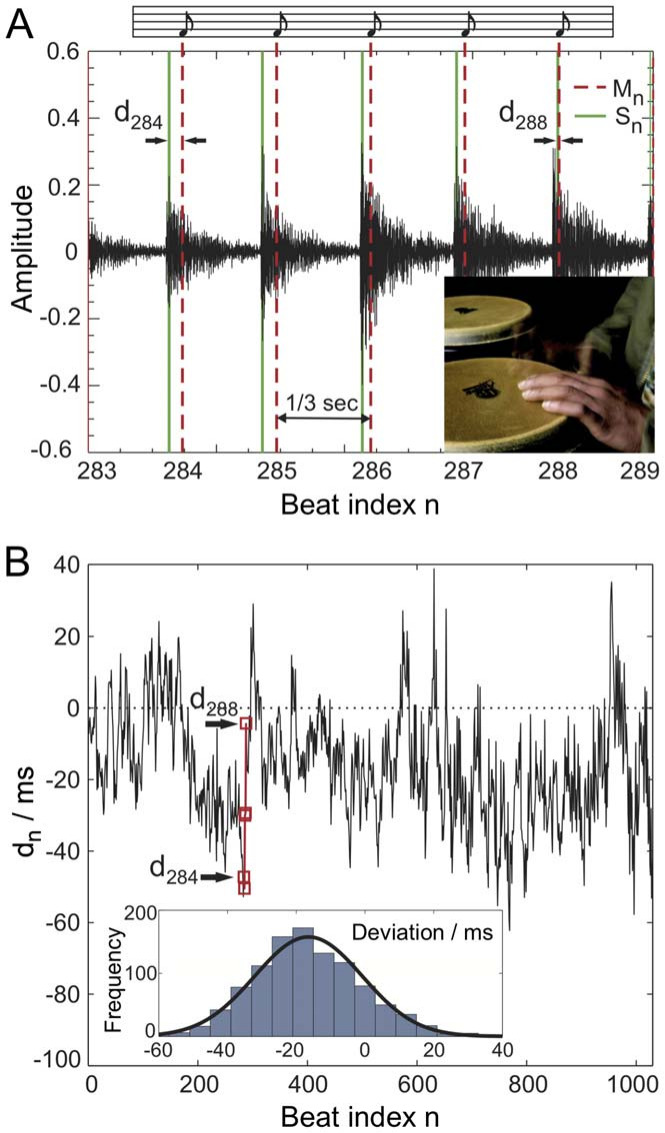
Demonstration of the presence of temporal deviations and LRC in a simple drum recording. A professional drummer (inset) was recorded tapping with one hand on a drum trying to synchronize with a metronome at 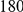 beats per minute (**A**). An excerpt of the recorded audio signal is shown over the beat index 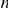 at sampling rate 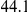 kHz. The beats detected at times 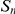 (green lines, see Methods) are compared with the metronome beats (red dashed lines). (**B**) The deviations 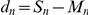 fluctuate around a mean of 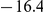 ms, i.e. on average the subject slightly anticipates the ensuing metronome clicks. Inset: The probability density function of the time series is well approximated by a Gaussian distribution (standard deviation 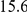 ms). Our main focus is on more complex rhythmic tasks, however (see Table 1). A detrended fluctuation analysis of {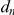} is shown in Fig. 2C (middle curve).

We analyzed the deviations from the exact beats for various combinations of hand, feet, and vocal performances, by both amateur and professional musicians. The data includes complex drum sequences from different musical bands obtained from a recording studio. While intentional deviations from an ideal rhythmic pattern play an important role in the interpretation of musical pieces, we focused here on the study of ‘natural’ (i.e. unintended intrinsic) deviations from a given rhythmic pattern of given complexity. In order to measure the deviations of human drumming from a rhythmic reference pattern we took a metronome as a reference and determined the temporal displacement of the recorded beat from the metronome click. A simple example of a recording is shown in Fig. 1: Here a test person had to follow the clicks of a metronome (presented over headphones) beating with one hand on a drum. Fig. 1A shows an excerpt of the audio signal and Fig. 1B the time series of deviations {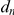}.

The recordings of beat sequences for several rhythmic tasks performed by humans are compiled in Table 1. In all recordings the subjects were given metronome clicks over headphones, a typical procedure in professional drum recordings. We used the grid of metronome clicks to compute the time series of deviations of beats and offbeats of complex drum sequences (tasks 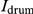). Each of the different drummers performed simultaneously with their feet (for bass drum and hi-hat) and hands. Furthermore we analyzed the recordings of vocal performances of four musicians (tasks 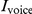), consisting of short rhythmic sounds of the voice according to a metronome at 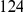 beats per minute (BPM). Short phonemes (such as [‘dee’]) were chosen to obtain well separated peaks in the envelopes of the audio signal. For comparison with less complex sensorimotor coordination tasks [4], [6], [8], [25], [28], [32] we have also included recordings of test subjects tapping with a hand on a drumhead (tasks 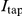). We recorded tapping at two different meters for each test subject, 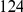 BPM and 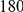 BPM, omitting so-called ‘glitches’, i.e. deviations larger than 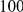 ms. These glitches occurred in less than 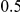
% and do not substantially affect the long term behavior we seek to quantify.

**Table 1:** Set of complex rhythmic tasks exhibiting LRC.

Rhythmic set	Task no.	Task description	Tempo (BPM)	Exponent 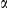	Exponent 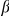
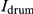	1	complex pattern	190	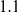	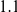
	2	complex pattern	132	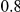	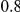
	3	periodic pattern	124	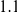	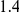
	4	periodic pattern	124	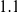	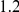
	5	periodic pattern	124	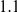	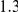
	6	tapping with stick	124	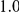	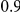
	7	tapping with stick	124	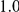	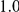
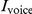	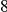	voice	124	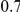	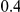
	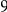	"	124	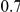	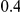
	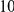	"	124	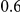	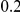
	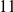	"	124	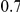	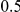
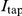	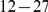	tapping with hand	180, 124	see Fig. 2B	see Fig. 2B

The exponents 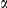 shown in the table were obtained using DFA and the exponents 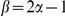 were computed via the PSD. In the set 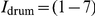 the complexity of rhythmic drum patterns decreases from tasks no. 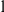 to 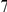. In tasks no. 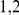 we analyzed real drum recordings that are taken from popular music songs, the two drummers are different persons. Tasks no. 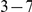 were performed by a third (different) drummer, where no. 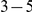 consisted of a short rhythmic pattern (that included beats and off-beats) repeated continuously by the drummer. We analyzed the fluctuations of both beats and off-beats (no. 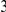) of the pattern and in addition we considered the fluctuations of the beats (task no. 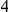) and off-beats (no. 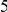) separately. See main text for a description of 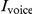 and 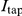.

We focused on the extraction of possible long-range correlations (LRC) from the recorded signals. A signal is called long-range correlated if its power spectral density (PSD) asymptotically scales in a power law, 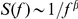 for small frequencies and 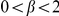. The power law exponent 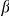 measures the strength of the persistence. The signal is uncorrelated for 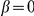 (white noise), while for 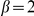 the time series typically originates from integrated white noise processes, such as Brownian motion. For 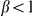 the Wiener Khinchin Theorem links the PSD to the autocorrelation function of the time series {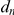} which also decays in a power law 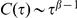. For the *superpersistent* case 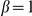 the correlation function does not decrease within the scaling regime whereas 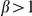 indicates instationarity of the time series.

We applied various methods that measure the strength of LRC in short time series, namely detrended fluctuations analysis (DFA) [21], [33], zero padding PSD method [34], and maximum likelihood estimation (MLE) (see Methods for details). DFA involves calculation of the fluctuation function 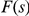 measuring the average variance of a time series segment of length 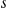. For fractal scaling one finds 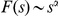, where 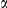 is the so-called Hurst exponent, which is a frequently used measure to quantify LRC [33]. The DFA exponent 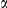 is related to the power spectral exponent 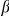 via 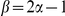 [35].

## Results

**Figure 2:**
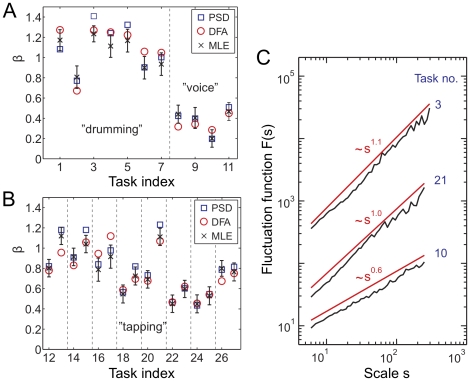
Evidence for long-range correlations in time series of deviations for the rhythmic tasks of **table 1**. The tasks correspond to complex drum sequences (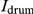) and rhythmic vocal sounds (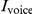) (**A**) and are compared to hand tapping on the drumhead of a drum (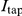) (**B**). The time series analysis reveals Gaussian 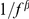-noise for the entire data set. The exponents 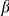 obtained by different methods, maximum likelihood estimation (incl. confidence intervals), detrended fluctuation analysis (DFA) and PSD show overall good agreement. Task indices separated by vertical dashed lines in Fig. 2B are recordings from different test subjects each recorded at two different metronome tempi: 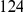 BPM (even index) and 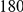 BPM (odd index). In (**C**) a DFA analysis for three representative time series is shown, corresponding to drumming a complex pattern where hands and feet are simultaneously involved (top), and tasks taken from 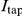 (middle) and 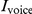 (bottom curve). The upper two curves are offset vertically for clarity. The curves clearly exhibit a power law scaling 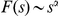 and demonstrate LRC in the time series. The total length of the time series is 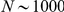.

We found LRC in musical rhythms for *all* tasks and subjects (summarized in Table 1) that were able to follow the rhythm for a sufficiently long time. Fig. 2C shows typical fluctuation functions for several time series of deviations {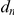}. The power law scaling indicates 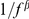-noise. The obtained power spectral densities display exponents 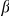 in a broad range, 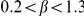 (Fig. 2 A–B). This strongly indicates that a (conceivable) unique universal exponent is very unlikely to exist (cf. the confidence intervals for 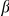). For the sets 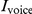 and 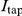 we recorded musicians and non-musicians with different musical experience ranging from laypersons to professionals. Our findings suggest that it is not possible to assign a correlation exponent (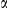 or 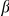) to a certain musician, as a single person may perform differently, which is quantified by both power law exponents 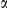 and 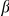 (see, e.g. tasks no. 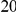 and 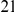 in Fig. 2B, which corresponds to beating on a drum at different metronome tempi). A systematic study of a possible dependence of the correlation exponents on the nature of the task would be interesting in itself but is beyond our focus here.

Although a musician does not necessarily intend to optimize synchrony with the metronome, the lowest values of the standard deviation of the time series were found for experienced musicians (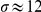 ms, average standard deviation 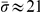 ms). We observed the trend that small values of 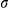 correlated with small values of 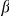 and therefore conjecture that on average, the more a person synchronizes with the metronome clicks, the lower the exponent 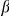. Hence, the degree of correlations in the interbeat time series would shrink with increasing external influence. In finger tapping experiments [8], [25] LRC were found even without the usage of a metronome [4], i.e., for weak external influence. On the other hand, in the extreme case where the test subject is triggered completely externally (as in reaction time experiments), LRC were entirely absent [4].

In order to probe nonlinear properties in the time series, we applied the magnitude and sign decomposition method [36], [37]. The analysis showed strong evidence for an absence of nonlinear correlations in the data (see Methods and Fig. S2). Thus the applied methods of analysis designed to characterize linear LRC are appropriate for the time series of deviations that we have studied here.

### Humanizing music

Do listeners prefer music the more the rhythm is accurate? In order to modify precise computer-generated music to make it sound more natural, professional audio software applications are equipped with a so-called ‘humanizing’ feature. It is also frequently used in post-processing conventional recordings. A humanized sequence is obtained by the following steps (see Methods for details): First, a musical sequence with beat-like characteristics is decomposed into beats [38], which are then shifted individually according to a random time series of deviations {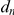}. The decomposed beats are finally merged again using advanced overlap algorithms.

**Figure 3:**
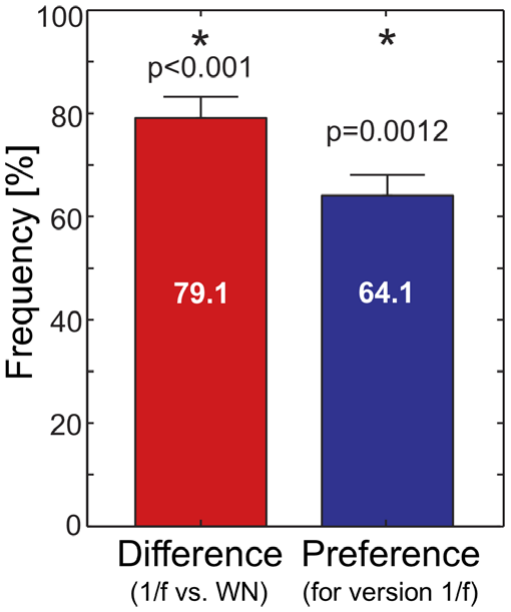
Perception analysis showing that 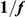 humanized music is preferred over white noise humanizing. The versions 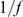 and WN (white noise) were compared by 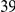 listeners. Two samples were played in random order to test subjects, all singers from Göttingen choirs, who were asked either one of two questions: *(1) “Which sample sounds more precise?”* (red bar) or *(2) “Which sample do you prefer?”* (blue bar) The answers to the first question provide clear evidence that listeners were able to perceive a difference between the two versions (t-test, 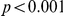). Furthermore, the 1/f humanized version was significantly preferred to the WN version (t-test, 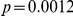).

We found that the humanizing tools of widely used professional software applications generally apply white noise fluctuations to musical sequences. Based on the above results we have studied the question whether musical perception can be influenced by humanizing using different noise characteristics. Therefore, a song was created and humanized with 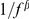-noise with 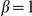 (*‘version* 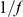 *’*), and white noise (‘*version WN*’), where the time series of deviations have zero mean and standard deviation 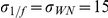 ms. Vocals remained unchanged, as well as all other properties of the versions such as pitch and loudness fluctuations, which have been shown to affect the auditory impression [1], [2]. Listeners were able to discriminate between the two versions, see Fig. 3. We observed a clear preference for the 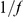 humanizing over the white noise humanized version, see the *audio example* in the Supporting Information where we compare the two versions.

### Rating of humanized music

Next, we describe our empirical analysis on the preference of listeners for 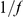 and WN (white noise) humanized music, cf. Fig. 3. Two segments of the song under investigation, which were about 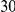 seconds long, were used. The two versions, 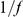 and WN of a segment were played three times to participants. Thus they listened to a total of six pairs (three times the pairs for the first segment, followed by pairs of the second segment). The order of the exact and the 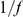 version was randomized across the six pairs, with each version being played the first in three out of six times. For each pair of segments participants were asked one of two questions: *(1) “Which sample sounds more precise?”* or *(2) “Which sample do you prefer?”* Half of the participants first made their preference judgments for all six pairs before they reheard the same six pairs and were asked which of the two versions sounded more precise. The second half of the participants received the same tasks in reversed order. No feedback was provided regarding which of the two versions was more precise.

In total 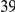 members of Göttingen choirs volunteered for the study; 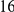 male and 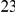 female (average age 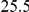 years, 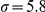). Participants were asked to assess their musical expertise on a scale from 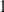 (amateur) to 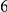 (professional). Participants rated their musical expertise on average as 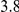 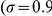). For both tasks (preference and precision) the relative frequency of choosing the 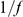 version was computed for each participant. The mean relative frequency with respect to preference for the 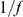 version was 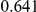 (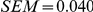). A 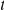-test revealed that these choices deviated significantly from chance (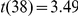, 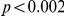), which indicates that participants clearly preferred the 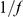 version over the white noise version. In addition they considered the 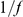 version to be more precise (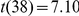, 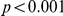) with frequency 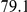
% (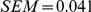).

## Discussion

This study provides strong evidence for LRC in a broad variety of rhythmic tasks such as hand, feet, but also vocal performances. Therefore these fluctuations are unlikely to be evoked merely by a limb movement. Another observation rather points to mechanisms of rhythmic timing (‘internal clocks’) that involve memory processes: We found that LRC were entirely absent in individuals who frequently lose rhythm and try to reenter following the metronome. The absence of LRC plays an important role as well in other physiological systems, such as heartbeat fluctuations during deep sleep [22]. While LRC in heartbeat intervals are reminiscent to the wake phase, they were found only in the REM phase of sleep, pointing to a different regulatory mechanism of the heartbeat during non-REM sleep.

Here, the loss of LRC may originate from a resetting of memory in the neurophysical mechanisms controlling rhythmic timing (e.g. neuronal ‘population clocks’, see [29], [30], [39] for an overview on neurophysical modeling of timing in the millisecond regime). In the other cases, the existence of strong LRC shows that these clocks have a long persistence even in the presence of a metronome. In cat auditory nerve fibers, LRC were found in neural activity over multiple time scales [12]. Also human EEG data [13] as well as interspike-interval sequences of human single neuron firing activity [14] showed LRC exhibiting power-law scaling behavior. We hypothesize that such processes might be neuronal correlates of the LRC observed here in rhythmic tasks.

In conclusion, we analyzed the statistical nature of temporal fluctuations in complex human musical rhythms. We found LRC in interbeat intervals as a generic feature, that is, a small rhythmic fluctuation at some point in time does not only influence fluctuations shortly thereafter, but even after tens of seconds. Listeners as test subjects significantly preferred music with long-range correlated temporal deviations to uncorrelated humanized music. Therefore, these results may not only impact applications such as audio editing and post production, e.g. in form of a novel humanizing technique [40], but also provide new insights for the neurophysical modeling of timing. We established that the favorable fluctuation type for humanizing interbeat intervals coincide with the one generically inherent in human musical performances. Further work must be undertaken to reveal the reasons for this coincidence.

## Methods

### Beat detection

Let the recording be given by the audio signal amplitude 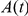. We define the occurrence of a sound (or beat) at time 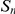 in the audio signal by
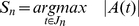
(1)where 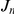 is the time interval of interest. Given the metronome clicks 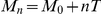, where 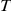 is the time interval between the clicks and 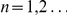, and given the sounds at times 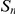, then the deviation 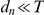 is obtained by the difference
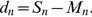
(2)The time series of interbeat intervals 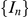 is given by 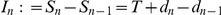. The beat detection according to Eq. 1 is suitable in particular for simple drum recordings due to the compact shape of a drum sound 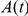 (see Fig. 1): The envelope of 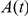 rises to a maximum value (“attack phase”) and then decays quickly (“decay phase”) [38]. Thus, if the drum sounds are well separated, a unique extremum 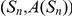 can be found. In contemporary audio editing software, typically the onset of a beat is detected [38], which is particularly useful when beats overlap. We used onset detection to find the temporal occurrences of sounds for humanizing musical sequences, as explained in the section “preparation of humanized music”.

We generalized definition Eq. 2 in order to consider deviations of a sequence from a complex rhythmic pattern (instead of from a metronome) as shown in Fig. S1.

### Audio example of humanized music

We created audio examples to investigate experimentally, whether there is a preference of listeners for LRC in music. Two segments of the pop song “Everyday, everynight”, which are about 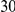 seconds long, were used. The song was created and humanized for our study in collaboration with Cubeaudio recording studio (Göttingen, Germany). An audio example consists of two different versions of the same segment of the song, played one after the other. You can listen to an audio example in the Supporting Information section (Audio S1). First, sample A, then sample B is played, separated by a 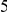 sec pause. The two samples differ only in the rhythmic structure, all other properties such as pitch and timbre are identical.

The first part (sample A) of the audio example (Audio S1) in the Supporting Information was humanized by introducing LRC (using Gaussian 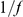-noise), while for sample B the conventional humanizing technique using Gaussian white noise was applied. While the samples used in the experiments on music perception contained also vocals, in this audio example vocal tracks were excluded for clarity.

### Preparation of humanized music

Here we provide details on how the music examples were prepared. For our study, a song was recorded and humanized in collaboration with Cubeaudio recording studio using the professional audio editing software ‘Pro Tools’ (Version HD 7.4). The song of 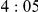 min. length has a steady beat in the eighth notes at 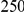 BPM, leaving a number of 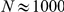 eighth notes in the whole song. First, the individual instruments were recorded separately, a standard procedure in professional recordings in music studios. This leads to a number of audio tracks while some instruments, such as the drum kit, are represented by several tracks. Then the beats which are supposed to be located at the eighth notes (but are displaced from their ideal positions) were detected for each individual track using onset detection [38] implemented in Pro Tools. The tracks are cut at the detected onsets of beats, resulting in 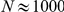 audio snippets for each track. Next, the resulting snippets were shifted onto their exact positions, which is commonly called ‘100% quantization’ in audio engineering. This procedure leads to an exact version of the song without any temporal deviations. At this stage humanizing comes into play. In order to humanize the song, we shifted the individual beats according to a time series of deviations 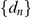, where 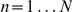. For example, if 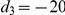 ms, then beat snippet no. 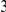 is shifted from its exact position by 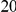 ms ahead (for all tracks). We generated two time series 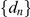, one is Gaussian white noise used for “version WN”, while the other consists of Gaussian 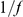 noise to generate “version 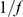
”. The snippets of the shifted beats are then merged in Pro Tools. The whole procedure was done for each audio track (hence, for each instrument). Finally, the set of all audio tracks is written to a single audio file.

### Probing correlations

A method tailored to probe correlation properties of short time series is detrended fluctuation analysis (DFA) which operates in the time domain [21], [33]. The integrated time series is divided into boxes of equal length 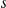. DFA involves a detrending of the data in the boxes using a polynomial of degree 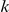 (we used 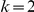 throughout this study). Thereafter the variance 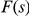 of the fluctuations over the trend is calculated. A linear relationship in a double logarithmic plot indicates the presence of power law (fractal) scaling 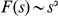. We considered scales 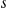 in the range 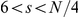, where 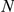 is the length of the time series. Once it is statistically established by means of PSD and DFA that the spectral density 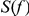 is well-approximated by a power law, we use the Whittle Maximum Likelihood Estimation (MLE) to estimate the exponent 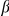 and determine confidence intervals. The MLE is applied to 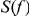 in the same frequency range as for the zero padding PSD method [34], which is 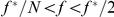, where 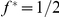 is the Nyquist frequency.

Moreover, we analyzed the data set with the magnitude and sign decomposition method that reveals possible nonlinear correlations in short time series [36], [37]. In a first step the increments of the original time series are calculated. Second, the increments are decomposed into sign and absolute value (referred to as magnitude) and its average is subtracted. Third, both time series are integrated. Finally, a DFA analysis is applied to the integrated sign and magnitude time series, and the scaling of the fluctuation function is measured. The magnitude series accounts for nonlinear correlations in the original error time series.

Fig. S2 shows four typical fluctuation functions (DFA) for their corresponding magnitude interbeat time series. The curves' slopes are close to 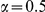 (bold black line). More precisely, 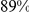 (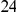 out of 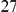) of the data set's magnitude exponents 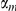 lie in the interval 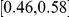; three values for 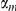 have values slightly above 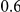 (each data set has less than 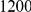 data points). Hence, the magnitude and sign decomposition method indicates a lack of nonlinear correlations in the original interbeat time series. Fig. S2 also displays the sign decompositions of the fluctuation functions. For small scales the curves have slopes below 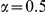, whereas those for large scales have slopes around 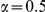. This behavior is representative for the whole data set. As an expected consequence, the sign decomposition shows small scale anticorrelations typically found in gait intervals [7], [24], together with a rather uncorrelated behavior on larger scales.

### Ethics statement

The study was reviewed and approved by the Ethics Committee of the Psychology Department of the University of Göttingen. The Committee did not require that informed consent was given for the surveys: return of the anonymous questionnaire was accepted as implied consent.

## Supporting Information

Figure S1Generalization of Eq. 2 in order to consider deviations of a complex rhythm from a complex pattern (instead of from a metronome), shown is a simple example. Beats at times 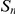 (red vertical lines) and an ideal beat pattern with beats at times 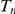 (black vertical lines) are compared. The resulting deviations read 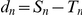. For illustration, the time is given in units of the length of a quarter note 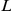, leading to the rhythmic pattern shown in the upper right corner.(TIFF)

Figure S2Probing nonlinear correlations in interbeat time series (the number in the legend denotes the task index, cf. table I of the article). Shown are plots of the root-mean-square fluctuation function 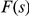 from second-order DFA analysis for (**A**) the integrated magnitude series, and for (**B**) the corresponding integrated sign series. The grey line has slope 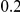 indicating anticorrelations. The black lines have slope 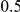 indicating no correlations.(TIFF)

Audio S1 Audio example: Pop song “Everyday, everynight”. First, the 1/f humanized version, then the white noise humanized version is played.
(MP3)
