# Correction: The Nature and Perception of Fluctuations in Human Musical Rhythms

**DOI:** 10.1371/annotation/f0a4c12c-ebef-4b55-9beb-2ca30749965e

**Published:** 2012-04-09

**Authors:** Holger Hennig, Ragnar Fleischmann, Anneke Fredebohm, York Hagmayer, Jan Nagler, Annette Witt, Fabian J. Theis, Theo Geisel

The authors mentioned a patent on the algorithm employed in this study and cited it as reference 40. In light of the journal's requirements, this information should also have been included under the Competing Interests section of the article. In addition, an EU patent on this algorithm is still pending. The authors would therefore like to revise the Competing Interests declaration to read as follows: Drs Hennig, Fleischmann, Theis, and Geisel have filed two patents related to the algorithm employed in this study. 

