# Supplementary figures and images for: The Nature and Perception of Fluctuations in Human Musical Rhythms
Source: PLoS One. 2011 Oct 26;6(10):e26457. doi: 10.1371/journal.pone.0026457 (PMC3202537; doi:10.1371/journal.pone.0026457)

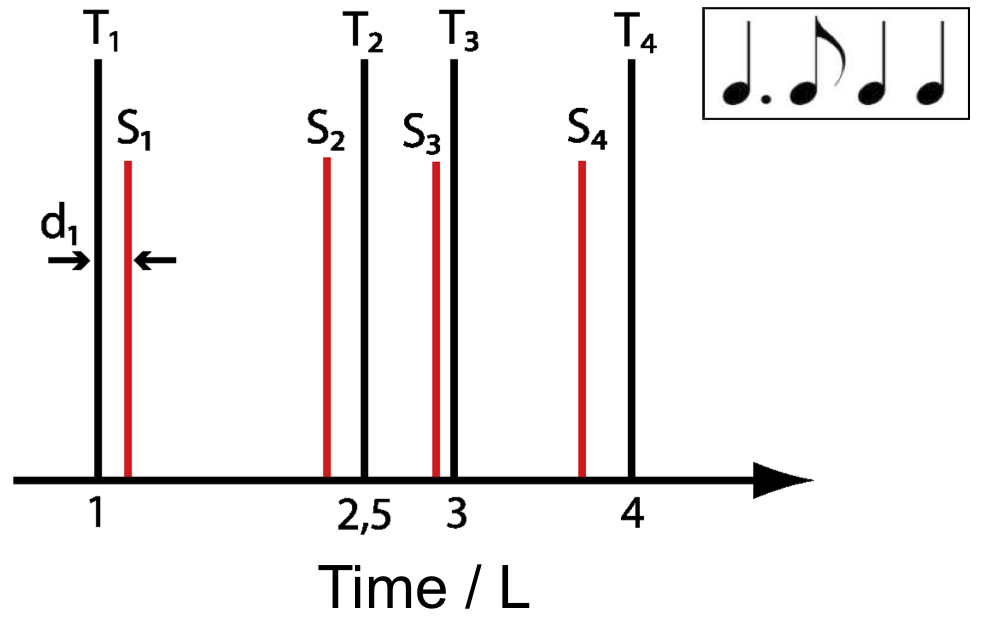

Supplement: Figure S1 — Generalization of Eq. 2 in order to consider deviations of a complex rhythm from a complex pattern (instead of from a metronome), shown is a simple example. Beats at times (red vertical lines) and an ideal beat pattern with beats at times (black vertical lines) are compared. The resulting deviations read . For illustration, the time is given in units of the length of a quarter note , leading to the rhythmic pattern shown in the upper right corner. (TIFF) [file pone.0026457.s001.tiff]

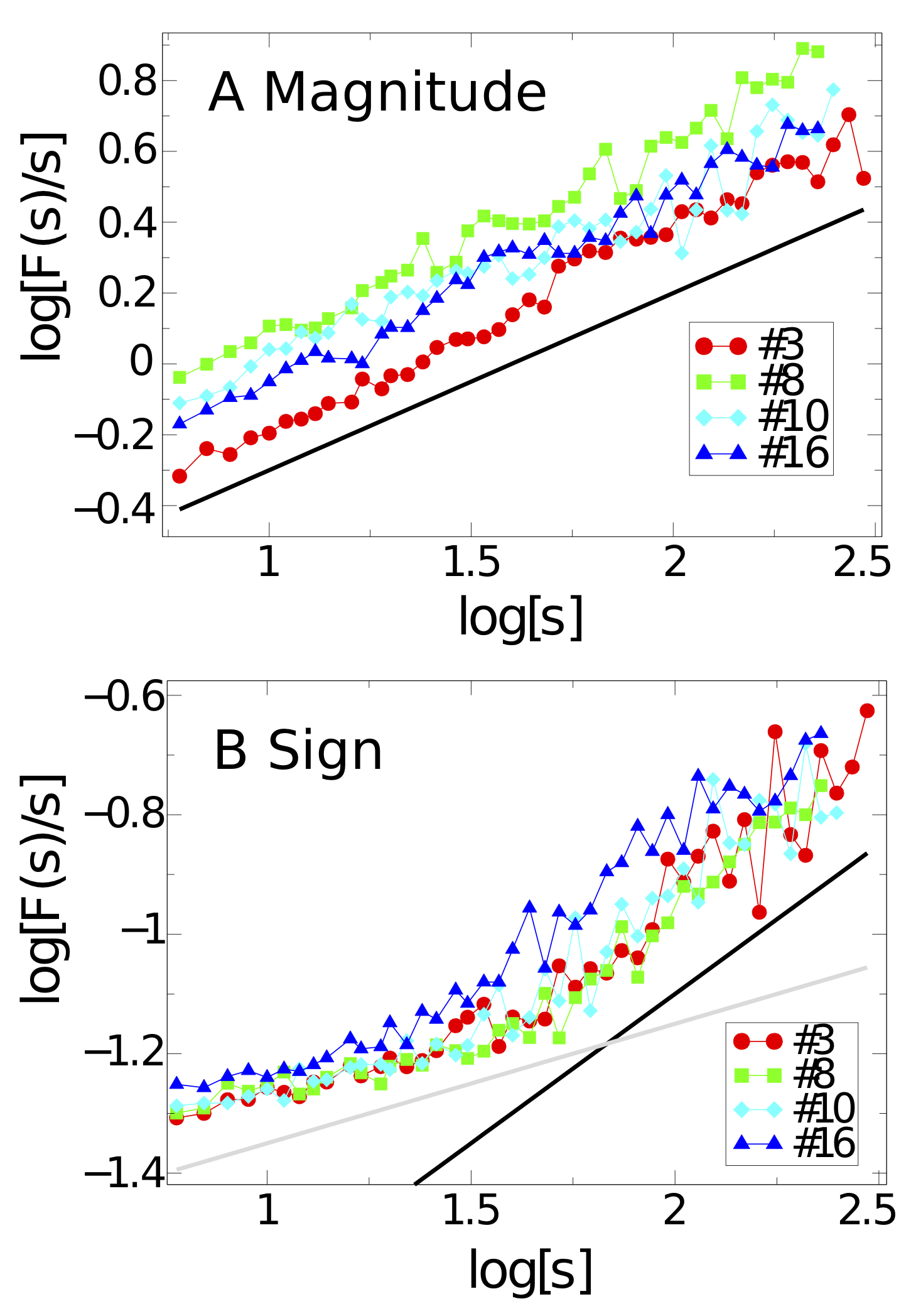

Supplement: Figure S2 — Probing nonlinear correlations in interbeat time series (the number in the legend denotes the task index, cf. table I of the article). Shown are plots of the root-mean-square fluctuation function from second-order DFA analysis for (A) the integrated magnitude series, and for (B) the corresponding integrated sign series. The grey line has slope indicating anticorrelations. The black lines have slope indicating no correlations. (TIFF) [file pone.0026457.s002.tiff]
